# Identification of biomarkers and immune infiltration in acute myocardial infarction and heart failure by integrated analysis

**DOI:** 10.1042/BSR20222552

**Published:** 2023-07-07

**Authors:** Wei Liu, Yuling Li, Yan Zhang, Su Li, Yuqiong Chen, Bing Han, Yao Lu

**Affiliations:** 1 Department of Cardiology, Xuzhou Central Hospital, Xuzhou Institute of Cardiovascular Disease, Xuzhou Clinical School of Nanjing Medical University, No. 199 Jiefang South Road, Xuzhou 221009, PR China; 2 Department of Ultrasonography, Xuzhou Central Hospital, Xuzhou Clinical School of Nanjing Medical University, No. 199 Jiefang South Road, Xuzhou 221009, PR China; 3 Department of Anesthesiology, Xuzhou Central Hospital, Xuzhou Clinical School of Nanjing Medical University, No. 199 Jiefang South Road, Xuzhou 221009, PR China; 4 Department of Cardiology, Shanghai Institute of Cardiovascular Diseases, Zhongshan Hospital, Fudan University, Shanghai, China; 5 Department of Cardiology, The Affiliated Suzhou Hospital of Nanjing Medical University, Suzhou Municipal Hospital, Gusu School, Nanjing Medical University, Suzhou, China

**Keywords:** acute myocardial infarction, diagnosis, expression profile, heart failure, immune cell infiltration, single cell sequencing

## Abstract

The mortality of heart failure after acute myocardial infarction (AMI) remains high. The aim of the present study was to analyze hub genes and immune infiltration in patients with AMI and heart failure (HF). The study utilized five publicly available gene expression datasets from peripheral blood in patients with AMI who either developed or did not develop HF. The unbiased patterns of 24 immune cell were estimated by xCell algorithm. Single-cell RNA sequencing data were used to examine the immune cell infiltration in heart failure patients. Hub genes were validated by quantitative reverse transcription-PCR (RT-qPCR). In comparison with the coronary heart disease (CHD) group, immune infiltration analysis of AMI patients showed that macrophages M1, macrophages, monocytes, natural killer (NK) cells, and NKT cells were the five most highly activated cell types. Five common immune-related genes (S100A12, AQP9, CSF3R, S100A9, and CD14) were identified as hub genes associated with AMI. Using RT-qPCR, we confirmed FOS, DUSP1, CXCL8, and NFKBIA as the potential biomarkers to identify AMI patients at risk of HF. The study identified several transcripts that differentiate between AMI and CHD, and between HF and non-HF patients. These findings could improve our understanding of the immune response in AMI and HF, and allow for early identification of AMI patients at risk of HF.

## Introduction

Genome-wide gene expression sequencing has been widely used to discover hub genes for cardiovascular disease [1]. In patients with acute myocardial infarction (AMI), immune cell infiltration plays an important role in both the acute phase and the reparative stage of myocardial damage [2]. Progression to heart failure (HF) after AMI is influenced by multiple factors, depending on the extent of myocardial damage, the time window of reperfusion, the presence of adverse left ventricular remodeling, and neuroendocrine regulation. Eventually, patients with AMI may develop heart failure, in which a severe decline in the systolic left ventricular function may lead to severe morbidity and mortality.

Several markers are known to be associated with the occurrence of HF after AMI, including troponin T (TNT) [3], a sensitive marker of myocardial damage, and brain natriuretic peptide (BNP) [4], a marker of HF. However, the discriminatory power of these markers to identify patients with AMI at risk for subsequent HF remains limited. Novel biomarkers like QSOX1 and PLBD1 [5], which predict left ventricular dysfunction after acute myocardial infarction, make up for the shortcomings of BNP to some extent. These indicators have fair diagnostic power to identify patients with AMI at risk for subsequent HF, but the discriminatory power remains modest. Therefore, new, reliable biomarkers for MI and HF after MI are needed.

Using novel techniques, such as xCell algorithm [6] and single cell sequencing [7], this study aims to achieve three objectives: first, to explore differences in immune cell infiltration between AMI and stable patients with coronary heart disease (CHD); second, to investigate explore immune cell infiltration between post-AMI HF and non-HF; and finally, to identify and validate novel biomarkers to predict AMI and AMI-induced HF.

## Methods

### Collection and preprocessing of publicly available expression datasets

**Figure 1:**
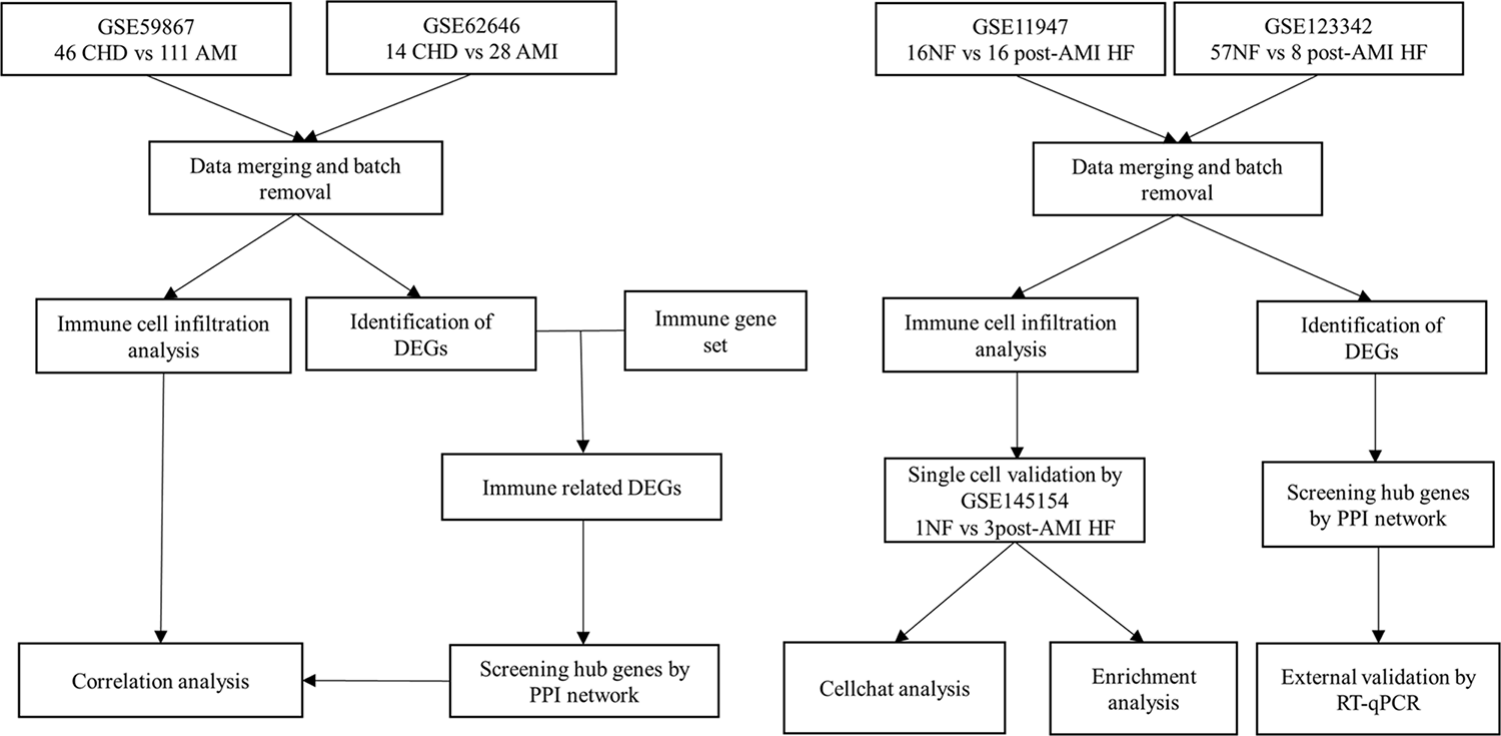
The step-by-step flowchart of this research

Five gene expression profile datasets (GSE59867, GSE62646, GSE145154, GSE11947, and GSE123342) were obtained from Gene Expression Omnibus (GEO) database (https://www.ncbi.nlm.nih.gov/geo/). The mRNA expression profiling datasets GSE59867 [8] and GSE62646 [9] both based on GPL6244 (Affymetrix Human Gene 1.0 ST Array) were downloaded from the GEO database (https://www.ncbi.nlm.nih.gov/geo/). GSE59867 and GSE62646 are detected by the same platform. This ensures the consistency of the sequencing process. GSE59867 contains peripheral blood mononuclear cells (PBMCs) from 111 patients with AMI and 46 CHD control. GSE62646 contains 28 AMI patients and 14 CHD control. The GSE59867 and GSE62646 datasets were merged and normalized using the ‘sva’ R package. To obtain the DEGs in different groups, we conducted DEGs analysis with normalized expression data using ‘limma’ R package (abs(log2FC)>0.5 and adjusted *P*-value <0.05). The mRNA expression profiling datasets GSE123342 [5] based on GPL17586 (Affymetrix Human Transcriptome Array 2.0) were downloaded from the GEO database (https://www.ncbi.nlm.nih.gov/geo/). The mRNA expression profiling datasets GSE11947 [10] based on GPL1947 (RNG-MRC_HU25k_STRASBOURG) were downloaded from the GEO database (https://www.ncbi.nlm.nih.gov/geo/). GSE123342 contains 65 AMI patients, of which 8 developed HF. GSE11947 contains 32 AMI patients, of which 16 developed HF. The GSE123342 and GSE11947 datasets were merged and normalized using the ‘sva’ R package. The two data sets use different Arrays, which may affect the reliability of the results, so further experimental verification is used. The mRNA expression profiling datasets GSE145154 [11] based on GPL20795 (Hiseq X Ten) was downloaded from the GEO database (https://www.ncbi.nlm.nih.gov/geo/). GSE145154 contains single cell sequencing of 3 HF patients and one healthy control. The expression value was preprocessed using the ‘normalize between arrays’ function in the ‘limma’ package [12]. The list of immune-related genes was downloaded from the ImmPort database (https://www.immport.org/shared/home). The step-by-step flowchart of this research was shown in Figure 1.

### Criteria of AMI and HF

Patients with AMI were enrolled in the different studies according to their respective criteria. Typically, all patients with suspected AMI underwent coronary angiography to confirm the diagnosis, followed by angioplasty of the infarct-related artery as necessary, which is considered the gold standard for diagnosis of myocardial infarction. Heart failure (HF) was defined as a reduction in left ventricular function to 40% or below during follow-up.

### Immune cell infiltration analysis by xCell algorithm

xCell [13] is a novel gene signature-based strategy used to infer variety immune and stromal cell types, and has been validated using extensive *in silico* simulations as well as immune phenotyping by cytometry. Applying xCell to the normalized data, portrayals of cellular heterogeneity landscape for cardiac tissue expression profiles can be acquired. Group boxplots were generated and compared using the Wilcoxon rank-sum test to determine significant differences between cell types, with a cutoff value of *P*<0.05. Correlation matrix of immune cell subtypes was constructed by corrplot package in R [14]. Spearman correlation coefficients were calculated and used for evaluating the strength of correlation.

### Estimation of enrichment pathway by single sample gene-set enrichment analysis (ssGSEA) algorithm

ssGSEA was introduced to quantify the relative enrichment score of specific pathways in a certain condition [15]. The specific feature gene panels for marking each immune pathway were curated from the ImmPort database. The relative abundance of each immune related pathway was represented by an enrichment score in ssGSEA analysis and normalized to unity distribution from 0 to 1. The biosimilarity of the enrichment pathway was estimated by multidimensional scaling (MDS) and a Gaussian fitting model.

### DEGs identifying

To obtain the DEGs in different groups, we conducted DEGs analysis with normalized expression data using ‘limma’ R package (abs[log2FC]>0.5 and adjusted *P*-value <0.05).

### Identification of hub genes in functional modules and crucial gene mining

We used STRING database (Protein–Protein Interaction Networks Functional Enrichment Analysis; http://string-db.org) to search for the protein–protein interaction (PPI) pairs of DEGs in the CHD and AMI groups. A PPI network map was constructed using Cytoscape software. Meanwhile, Cytohubba identified the most important module of the network map. The criteria for analysis was MCC (Maximal Clique Centrality) score >15. Hub genes were excavated by setting the degrees.

### Single-cell RNA data processing

In our single-cell sequencing analysis, we utilized raw data from GSE145154 was used and analysed using the package of Seurat [16] in R (version: 4.0.3) with R studio (version:1.3.1903) [17]. Cells were filtered with the criteria of >20% mitochondria related genes or expressing >6,000 genes expressed. Cells expressing <300 genes and genes expressed in <3 cells were filtered out. A total of 20,329 cells were included for further analysis, and variable features of each sample were analyzed after normalization. The FindVariableFeatures was used to identify the 10 most highly variable genes. The data of cells were clustered into 10 cell populations using FindClusters (resolution = 0.5). We performed various methods for cell clustering reduction, including UMAP, t-SNE, and PCA. For cell population annotation, we used ‘singleR’ package [18]. Then we used CellChat [19], which is a tool that can quantitatively infer and analyze intercellular communication networks from single-cell RNA-sequencing (scRNA-seq) data. Using network analysis and pattern recognition approaches, CellChat predicts major signaling inputs and outputs for cells and how those cells and signals coordinate for functions. Through manifold learning and quantitative contrasts, CellChat classifies signaling pathways and delineates conserved and context-specific pathways across different datasets.

### Biological enrichment analysis

Biological signaling pathways reflect the biological changes. The Gene Ontology (GO) and Kyoto Encyclopedia of Genes and Genomes (KEGG) pathways are the two commonly used pathway gene sets [20]. The differential analysis was set to adjust the *P*-value <0.01 as the cutoff criterion. The biological function for DEGs was analyzed by GO enrichment analysis using the R package ‘clusterProfiler’ [21].

### Summarize the protein expression levels of immune-related DEGs in peripheral blood

The Human Protein Atlas (HPA) database [22] was used to find the expression of immune-related DEGs in each type of immune cell, and the relative expression ratio of genes in each immune cell was obtained by percentage histogram.

### Patient and blood collection

We retrospectively selected 60 patients diagnosed with myocardial infarction on admission, whose peripheral blood were stored in the biobank of Xuzhou Central Hospital. After one month of follow-up, patients were divided into HF group (*n*=20, LVEF < 50%) and non-HF group (*n*=40, LVEF > 50%). Blood of patients was stored in a biosafe refrigerator at -80°C prior to use. The work was approved by the ethical committee of The Affiliated XuZhou Hospital of Nanjing Medical University, and an IRB (Institutional Review Board) approval (XZXY-LY-20161007-043) was given prior to this study.

### RNA extraction and quantitative real-time PCR (RT-qPCR)

RNA was extracted from blood by Trizol (Invitrogen, Carlsbad, CA, U.S.A.), according to the protocols of manufacturer. Spectrophotometer (NanoDrop‐2000, Thermo Fisher Scientific) was to inspect the quantity and quality of RNA. The steps for PCR were performed as previously described [23]. The sequences of primers used in the study are shown in Supplementary Table S1. The relative expression was calculated using the following equation: relative gene expression = 2∧ ^(ΔCtsample − ΔCtcontrol)^. All samples were measured in triplicate.

## Results

### Immune cell landscape in AMI after merging GSE59867 and GSE62646 datasets

**Figure 2:**
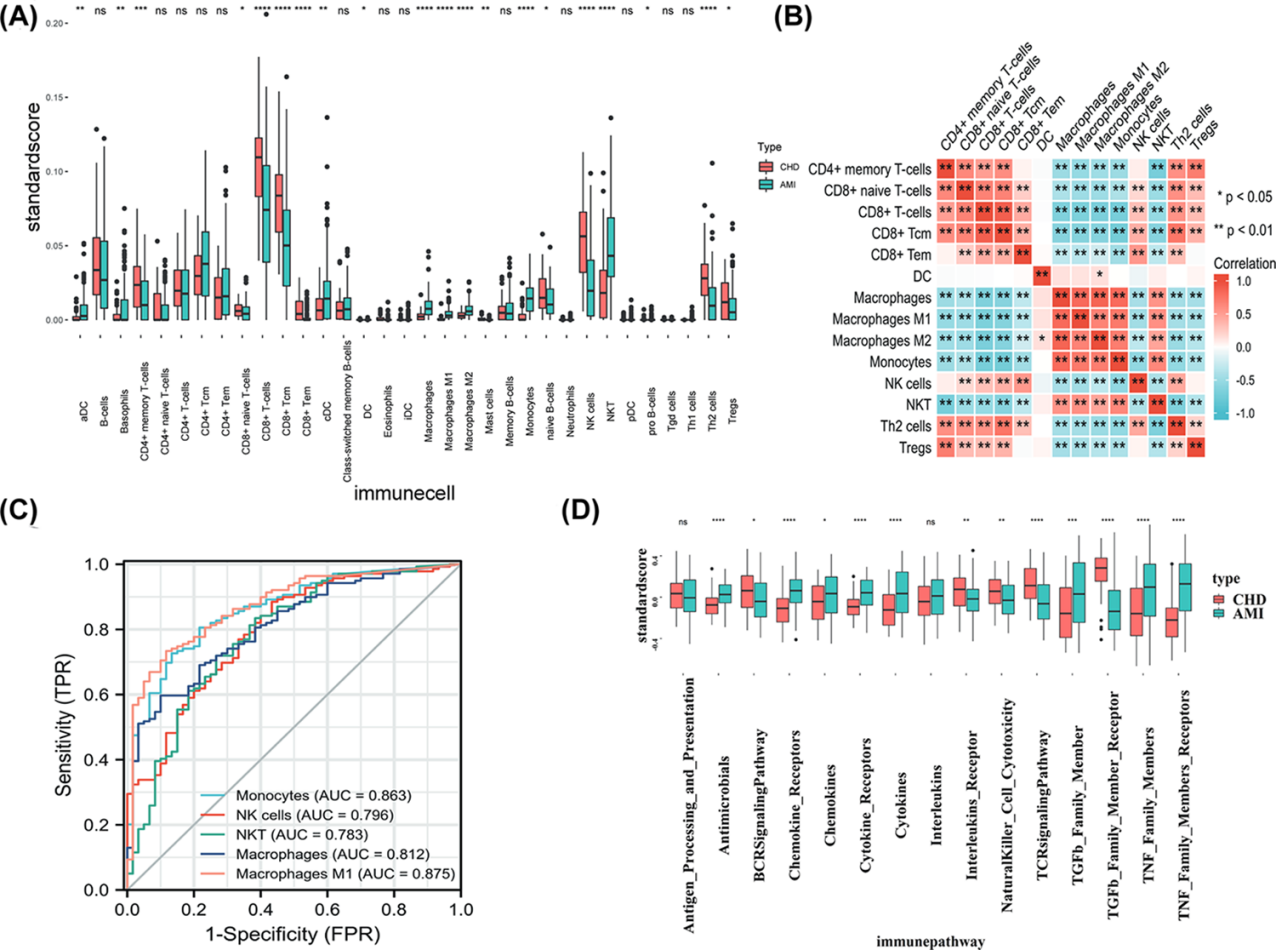
Immune cell landscape in AMI patients after merging GSE59867 and GSE62646 datasets (**A**) The differences in immune cell enrichment scores between the acute myocardial infarction (AMI) and coronary heart disease (CHD) group by using xCell algorithm (* means *P*<0.05, ** means *P*<0.01, *** means *P*<0.001, **** means *P*<0.0001). (**B**) Correlation between 14 significantly changed immune cell was visualized with a heatmap (*means *P*<0.05, ** means *P*<0.01). (**C**) An area under the ROC curve (AUC) of top five immune cell abundance in merged cohort. (**D**) Pathway enrichment analysis by ssGSEA between acute myocardial infarction (AMI) and coronary heart disease (CHD) group in merged dataset (* means *P*<0.05, ** means *P*<0.01, *** means *P*<0.001, **** means *P*<0.0001).

The GSE59867 and GSE62646 datasets were merged and normalized using the ‘sva’ R package, and finally 60 CHD and 139 AMI patients were included. We first compared the immune cell fractions of 139 patients with AMI on day 1 and 60 patients with CHD using the xCell algorithm and the RNA expression profile in PBMCs. The proportion results of 33 kinds of immune cells obtained from xcell analysis of integrated data sets were described in Supplementary Table S2. Immune infiltration analysis showed a higher level of activated dendritic cells (aDC), basophils, conventional DCs (cDCs), macrophages, M1 macrophages, M2 macrophages, monocytes, and NKT cells, as well as a lower level of CD4+ memory T cells, CD8+ naïve T cells, CD8+ T cells, CD8+ central memory T cells (CD8+ Tcm), CD8+ effecter memory T cells (CD8+ Tem), naïve B cells, NK cells, Th2 cells, and Tregs in AMI compared with the CHD group (Figure 2A). Correlation between 14 immune cell enrichment score was visualized with a heatmap (Figure 2B). Macrophages M1, Macrophages, monocytes, NK cells, and NKT cells were top five changed immune cells between AMI and CHD in merged datasets (Figure 2C). Pathway enrichment analysis by ssGSEA showed higher score of antimicrobials, chemokine receptors, chemokines, cytokine receptors, cytokines, TGF-β family member, TNF family members, TNF family members receptors, as well as lower score of BCR signaling pathway, interleukins receptors, natural killer cell cytotoxicity, TCR signaling pathway, and TGF-β family member receptor pathway in AMI group compared with the CHD group (Figure 2D).

### Identification of common immune-related DEGs in AMI after merging GSE59867 and GSE62646 datasets

**Figure 3:**
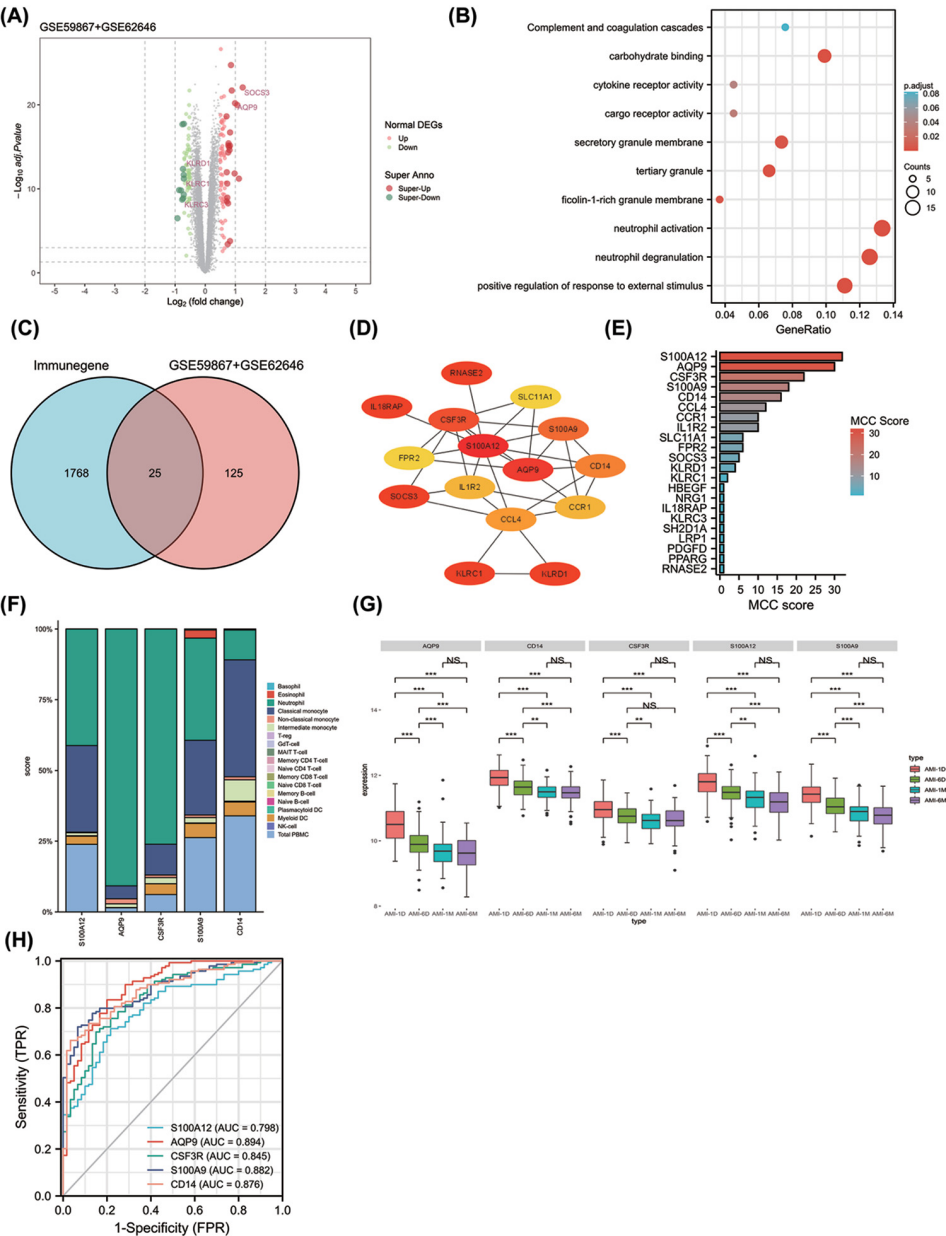
Identification of common immune-related DEGs and predictive performance in merged GSE59867 and GSE62646 datasets (**A**) Differentially expressed genes between acute myocardial infarction and CHD samples in merged dataset (GSE59867+GSE62646). (**B**) Gene Ontology (GO) enrichment analysis were visualized with bubble plots. (**C**) The 25 common immune-related DEGs in merged dataset were found by intersecting common DEGs with genes from ImmPort database. (**D**) PPI network of DEGs using STRING and visualized it using Cytoscape software. (**E**) MCC (Maximal Clique Centrality) score of the DEGs was visualized with histogram plot. (**F**) Expression of five hub immune-related DEGs in each type of immune cell by the Human Protein Atlas (HPA) database. We downloaded the expression levels of five genes from the ‘immune cell’ module in HPA database, and showed the gene expression levels at the protein level of each immune cell by percentage histogram. (**G**) The expression levels of five hub immune-related genes from day 1 to 6 months of PBMC of AMI patients (** means *P* <0.01, *** means *P* <0.001). (**H**) An area under the ROC curve (AUC) of five hub immune-related genes in merged dataset.

The GSE59867 and GSE62646 datasets were merged and normalized using the ‘sva’ R package, and finally 60 CHD and 139 AMI patients were included. A difference analysis on the integrated data was conducted and was described in Supplementary Table S3. A total of 150 DEGs were obtained: 80 genes were significantly up-regulated and 70 genes were significantly down-regulated (Figure 3A). Gene Ontology enrichment analysis found DEGs are involved in neutrophil activation, neutrophil degranulation and positive regulation of response to external stimulus (Figure 3B). These findings suggest that immune response is significantly activated in AMI patients. To identify the most important immune genes in the acute phase of AMI, we first downloaded 1793 validated immune-related genes from the Immport Database. The common immune-related DEGs in merged datasets (GSE59867 and GSE62646) were found by intersecting common DEGs with genes from ImmPort database. As a result, we identified 25 common immune-related genes associated with AMI (Figure 3C). We established a PPI network of DEGs using STRING and visualized it using Cytoscape (Figure 3D). We also analyzed modules using the Cytoscape plugin and cytohubba, and we identified the top five genes (S100A12, AQP9, CSF3R, S100A9, and CD14) with MCC scores >15 (Figure 3E). The expression levels of S100A12, AQP9, CSF3R, S100A9 and CD14 were significantly upregulated in AMI patients compared with CHD patients. To investigate the expression of the five immune-related DEGs in each type of immune cell, we utilized the Human Protein Atlas (HPA) database. Our findings revealed that S100A12, AQP9, CSF3R, and S100A9 were most expressed in neutrophil while CD14 was most expressed in classical monocyte (Figure 3F).

### Changes of expression levels of co-immune-related DEGs in AMI patients and predictive performance in merged dataset

Our analysis revealed that the expression levels of all five genes exhibited a sustained decrease from day 1 to 1 month after the onset of myocardial infarction, but no further decrease in gene expression was observed during 1 month to 6 months in GSE59867 dataset (Figure 3G). S100A12, AQP9, CSF3R, S100A9, and CD14 was identified as the potential biomarkers with an area under the ROC curve (AUC) of 0.798, 0.894, 0.845, 0.882, and 0.876 in merged dataset (GSE59867+GSE62646) (Figure 3H).

### Correlation between the immune-related core DEGs and immune cell infiltration in merged dataset

**Figure 4:**
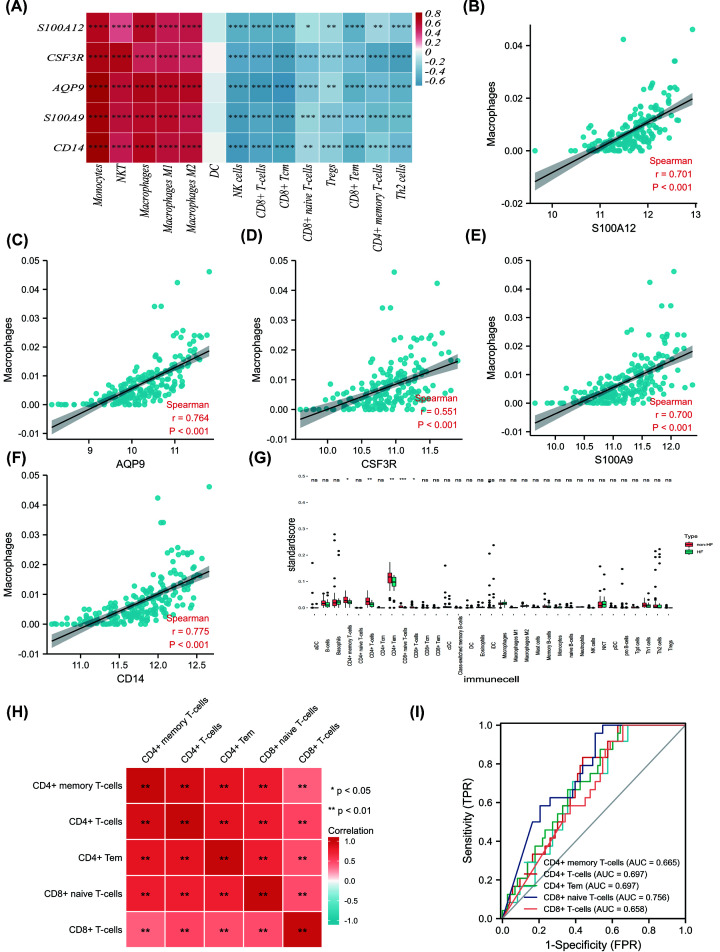
Correlation between the immune-related core DEGs and immune cell infiltration in AMI patients and immune cell landscape between non-HF and post-AMI HF patients after merging datasets (GSE11947 and GSE123342) (**A**) Correlation between the immune-related hub DEGs and immune cell infiltration score in merged dataset was visualized with heatmap. immune cell infiltration score was gained by xCell algorithm above. (**B–F**) Correlation between five hub immune-related genes (S100A12, AQP9, CSF3R, S100A9, and CD14) and macrophage cell infiltration proportion gained by xCell algorithm above. (**G**) The differences in immune cell abundance between the non-HF and HF group in merged dataset (GSE11947 and GSE123342) by using xCell algorithm (* means *P*<0.05, ** means *P*<0.01, *** means *P*<0.001, **** means *P*<0.0001). (**H**) Correlation between five significantly changed immune cell was visualized with a heatmap (* means P<0.05, ** means P<0.01). (**I**) An area under the ROC curve (AUC) of five immune cell abundance in merged dataset.

To further explore the correlation between the immune-related core DEGs and immune cell infiltration, a correlation heatmap was established (Figure 4A). A significant positive correlation was observed between S100A12, AQP9, CSF3R, S100A9, CD14 expression level and the infiltration score of monocytes, NKT cells, macrophages, M1 macrophages, M2 macrophages, while a significant negative correlation was found between S100A12, AQP9, CSF3R, S100A9, CD14 expression level and the infiltration score of NK cells, CD8+ Tcm, CD8+ naïve T cells, Tregs, CD8+ Tem, CD4+ memory T cells, Th2 cells. Significant positive correlation was found between all five genes and macrophage infiltration score (Figure 4B-F).

### Immune cell landscape of blood transcriptome between non-HF and post-AMI HF patients after merging datasets (GSE11947 and GSE123342)

To further investigate the differences of transcriptome between heart failure and non-heart failure after AMI, The GSE11947 and GSE123342 datasets were merged and normalized using the ‘sva’ R package, and finally 73 non-HF and 24 HF patients were included. We used the xCell analysis to compare the difference in immune infiltration between the two groups by merging datasets (GSE11947 and GSE123342) (Figure 4G). The proportion results of 33 kinds of immune cells obtained by xCell analysis of integrated data sets were described in Supplementary Table S4. When comparing HF patients with non-HF patients, we observed lower levels of CD4+ memory T cells, CD4+ T cells, CD4+ central memory T cells, CD8+ naive T cells, and CD8+ T cells. Correlation between five changed immune cell was visualized with a heatmap (Figure 4H). CD4+ memory T cells, CD4+ T cells, CD4+ central memory T cells, CD8+ central memory T cells, and CD8+ T cells were top five changed immune cell between HF patients and non-HF patients in merged datasets (Figure 4I).

### Further demonstration of immune microenvironment in peripheral blood of ischemic heart failure

**Figure 5:**
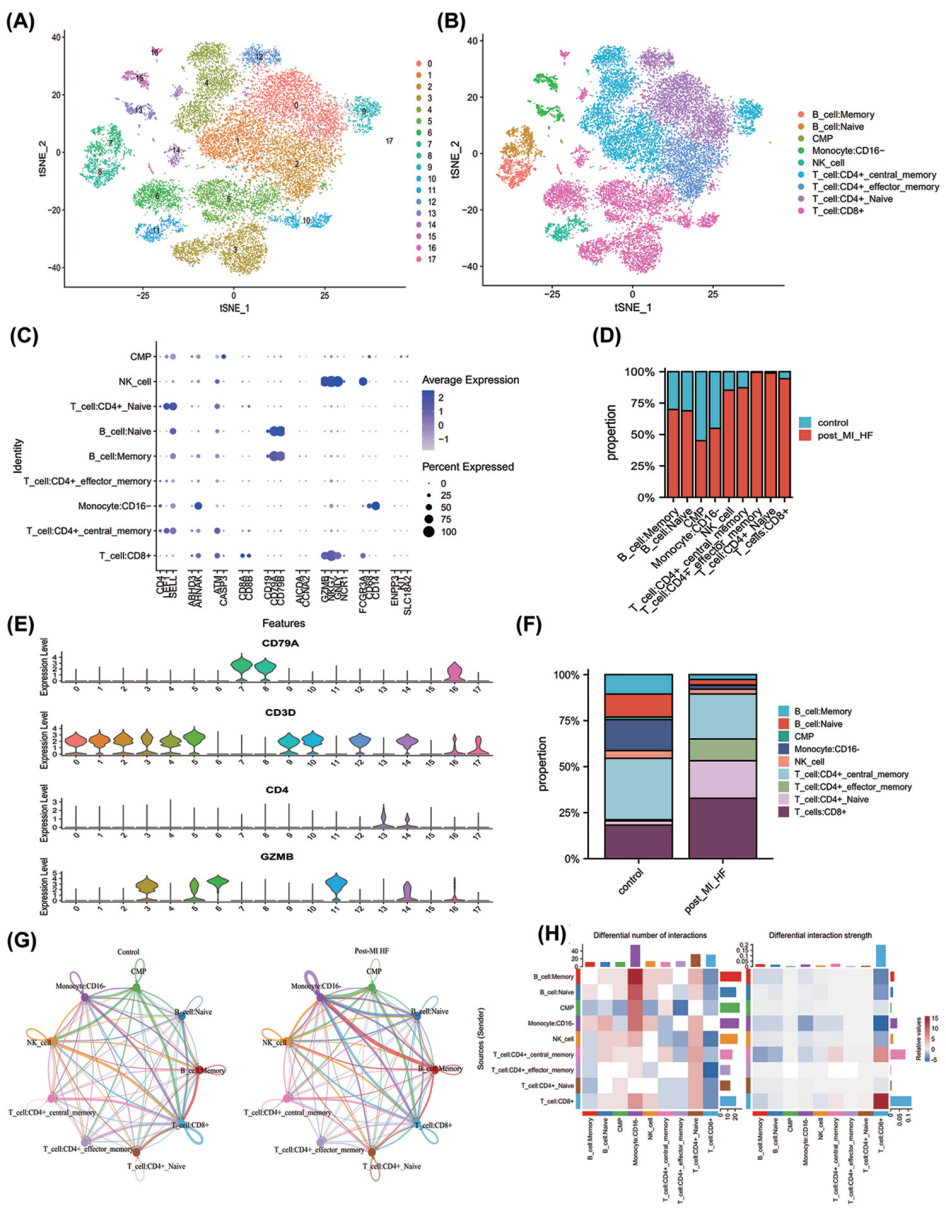
Further demonstration of immune microenvironment in peripheral blood of ischemic heart failure (**A,B**) 18 cell clusters in single cell sequencing dataset GSE145154 was calculated by ‘Seurat’ package (resolution = 0.5), and using ‘singleR’ package, we classified 18 cell clusters into 9 cell populations automatically. (**C**) The markers of immune cells markers in 9 cell population. (**E**) The markers of T-cell specific markers in 18 clusters. (**D,F**) The proportion of multiple immune cell subsets in health and patients with ischemic heart failure. (**G,H**) Immune cell intersection established by ‘Cellchat’ R package between control and heart failure group.

In addition to bulk sequencing analysis, we analyzed the immune microenvironment of ischemic heart failure with single cell sequencing dataset GSE145154. We further clustered immune cell populations into 18 clusters (Figure 5A) and classified them into 9 cell populations using ‘single R’ package, which were memory B cells, naïve B cells, Common Myeloid Progenitors (CMP), CD16- monocytes, NK cells, CD4+ central memory T cells, CD4+ effector memory T cells, CD4+ naïve T cells, mast cells, and NK cells (Figure 5B). The markers of immune cells and T-cell specific markers are visualized in Figure 5C,E. Compared with control group, the proportion of multiple immune cell subsets varied significantly in patients with ischemic heart failure (Figure 5D,F). The details of these variations were described in Supplementary Table S5. Patients with HF had lower a proportion of memory B cells, naïve B cells, CD16- monocytes and CD4+ central memory T cells, but higher proportion of CD4+ effector memory T cells, naïve CD4+ T cells and CD8+ T cells compared with control group. The results were consistent with the analysis by bulk sequencing analysis.

In order to explain changes in immune cell intersection in the heart failure group, we performed cell interaction analysis by using the ‘Cellchat’ R package. By analyzing the relative changes of the expression level of receptor and ligand, the interaction between different cells was simulated. Compared with control group, patients with heart failure had more interactions between memory B cell and CD16- monocytes, while less interactions between effector memory T cells and CD8+ T cells (Figure 5G,H). Pathway analysis by ‘Cellchat’ showed that NCAM, IL16, SELL, MK, PARs, SEMA4, CCL, MHC-II pathway were only enriched in the heart failure group (Supplementary Figure S1A). Through differential analysis, we identified differential gene sets in four significantly altered T cells in the HF group. An upset plot was used to find an intersection of four gene sets, which resulted in 42 common DEGs between CD4+ central memory T cells and CD8+ T cells (Supplementary Figure S1B). Subsequently, we conducted enrichment analysis for the DEGs of four kinds of immune cells (Supplementary Figure S1C–F), revealing that the ribosome pathway was enriched in all four types of immune cells.

### Identification and Validation of five hub DEGs in HF after AMI using RT-qPCR

**Figure 6:**
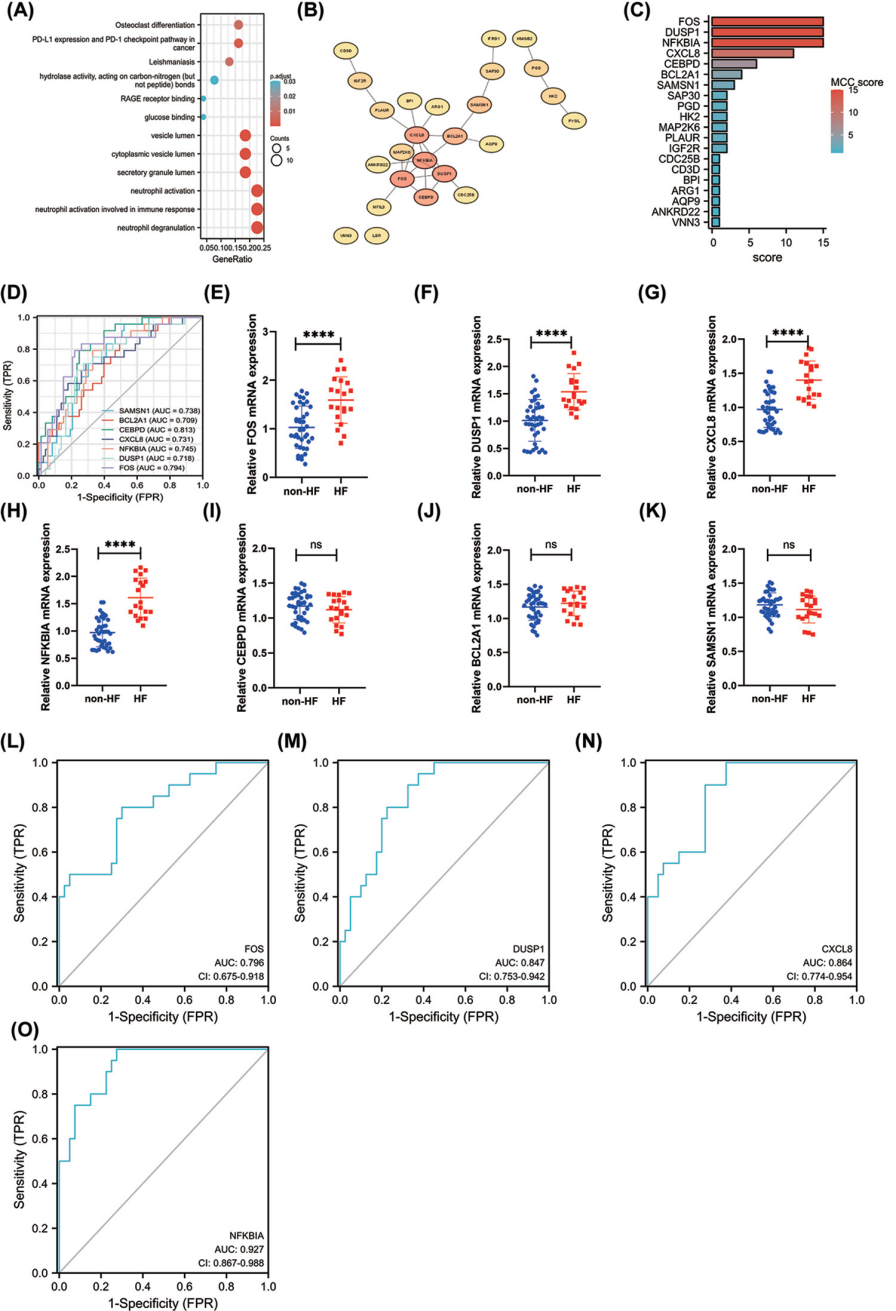
Identification and Validation of five hub DEGs in HF after AMI using RT-qPCR (**A**) Gene Ontology enrichment analysis of DEGs between HF and non-HF group merged dataset (GSE11947 and GSE123342). (**B**) PPI network of DEGs using STRING and visualized it using Cytoscape software. (**C**) MCC (Maximal Clique Centrality) score of the DEGs was visualized with histogram plot. (**D**) An area under the ROC curve (AUC) of seven hub DEGs to distinguish HF with non-HF patients in merged datasets with the AUC value >0.7. (**E–K**) Gene expression changes (FOS, DUSP1, CXCL8, NFKBIA, CEBPD, BCL2A1, and SAMSN1) were investigated in HF and non-HF on day 1 after AMI using RT-qPCR (**** means *P* <0.0001). (**L–O**) ROC curves for FOS, DUSP1, CXCL8, and NFKBIA; AUC, area under the curve; ROC, receiver operating characteristic.

We then compared RNA expression in patients with AMI who did or did not develop subsequent HF. A total of 59 DEGs were obtained between HF and non-HF patients in merged dataset (GSE11947 and GSE123342) and were described in Supplementary Table S6: 56 genes were significantly up-regulated and 3 genes were significantly down-regulated. Biological process of Gene Ontology enrichment analysis found DEGs are involved in PD-L1 expression and PD-1 checkpoint pathway in cancer, neutrophil activation, neutrophil activation involved in immune response, and neutrophil degranulation (Figure 6A). We established a PPI network of DEGs using STRING and visualized it using Cytoscape (Figure 6B). We also analyzed modules using the Cytoscape plugin and cytohubba module and identified the top 20 genes (Figure 6C). FOS, DUSP1, CXCL8, NFKBIA, CEBPD, BCL2A1, and SAMSN1 were top seven DEGs to distinguish HF with non-HF patients in merged datasets with the AUC value >0.7 (Figure 6D). FOS, DUSP1, CXCL8, NFKBIA, CEBPD, BCL2A1, and SAMSN1 were all up-regulated in blood of HF patients compared with non-HF patients. To further verify the effectiveness of core genes, we performed RT-qPCR in peripheral blood of patients with and without heart failure after MI. The baseline data of patients was presented in Supplementary Table S7. Using RT-qPCR, we confirmed that FOS, DUSP1, CXCL8, and NFKBIA were significantly up-regulated in HF (*n*=20) compared with non-HF (*n*=40) group in validation dataset, whereas CEBPD, BCL2A1, and SAMSN1 were not (Figure 6E–K). To investigate the value of FOS, DUSP1, CXCL8, and NFKBIA as biomarkers of HF, the ROC analysis was performed on the RT-qPCR data from patients with HF and non-HF patients. The analysis showed a good predictive accuracy of these markers (Figure 6I–O). FOS, DUSP1, CXCL8, and NFKBIA were identified as the potential biomarkers with an area under the ROC curve (AUC) of 0.796 (95% CI: 0.675–0.918), 0.847 (95% CI: 0.753–0.942), 0.864 (95% CI: 0.774–0.954), and 0.927 (95% CI: 0.867–0.988), respectively.

## Discussion

In this study, we aimed to explore the immune changes in patients with AMI and subsequent heart failure, with the goal of identifying new markers that could improve risk stratification. We have identified and characterized transcriptomic signatures and pathways associated with AMI based on gene expression analysis of PBMCs. Our results showed that a significant number of immune related genes exhibit altered expression during the acute phase of myocardial infarction. Hub immune-related genes associated with AMI are activated in day 1 to 1 month and gradually stabilized during 1–6 months. We report a clear difference in immune infiltration between patients with AMI and CHD, as well as AMI induced HF and non-HF patients. S100A12, AQP9, CSF3R, S100A9, and CD14 were identified as potential biomarkers to discriminate AMI from the control individuals. We also identified potential biomarkers, including FOS, DUSP1, CXCL8, and NFKBIA, that could differentiate between AMI-induced HF and non-HF patients.

The results of our gene enrichment analysis of DEGs associated with AMI demonstrated that the enriched pathways generally involved inflammation and immune response pathways, such as neutrophil activation. These findings are in line with the well-established and crucial pathophysiological role of the inflammatory response in AMI [24]. The significance of the immune system for cardiac repair after AMI is important, which has been confirmed to influence various repair processes.

We utilized the xCell algorithm to evaluate the types of immune cell infiltration in samples from patients with AMI and CHD samples, as well as AMI induced HF and non-HF patients with AMI. As a result, many types of immune cells were found to be involved in AMI and AMI induced HF. Immune infiltration analysis showed a higher level of activated dendritic cells (aDC), basophils, conventional DCs (cDCs), macrophages, M1 macrophages, M2 macrophages, monocytes, and NKT cells, as well as a lower level of CD4+ memory T cells, CD8+ naïve T cells, CD8+ T cells, CD8+ CD8+ Tcm, CD8+ Tem, naïve B cells, NK cells, Th2 cells and Tregs in the AMI group when compared to the CHD group. CD4+ memory T cells, CD4+ T cells, CD4+ central memory T cells, CD8+ central memory T cells, and CD8+ T cells may contribute to AMI induced heart failure. A significant positive correlation was observed between S100A12, AQP9, CSF3R, S100A9, CD14 expression level and the infiltration score of monocytes, NKT cells, macrophages, M1 macrophages, M2 macrophages, while a significant negative correlation was found between S100A12, AQP9, CSF3R, S100A9, CD14 expression level and the infiltration score of NK cells, CD8+ Tcm, CD8+ naïve T cells, Tregs, CD8+ Tem, CD4+ memory T cells, Th2 cells. Moreover, FOS, DUSP1, CXCL8, and NFKBIA were identified as four potential biomarkers to distinguish HF with non-HF patients. These results suggests a broad association between peripheral neutrophil infiltration or activation and heart failure [25]. Our present findings have demonstrated that several types of infiltrating immune cells play vital roles in AMI and AMI-induced HF, and should therefore be the focus of future research.

Infiltration of T cells and transformation into proinflammatory phenotypes has been shown to promote the progression of myocardial fibrosis and heart failure [26]. However, the origin and function of infiltrating T cells have not been clearly defined. By analysis of PBMCs from non-HF and post AMI HF patients, we found patients with HF had a higher proportion of CD4+ effector memory T cells, naïve CD4+ T cells and CD8+ T cells compared with controls. Furthermore, inflammatory-related pathways (IL16, CCL, MHC-II) were found to be activated in the heart failure group. Previous studies have reported that interleukin-16 promotes cardiac fibrosis and myocardial sclerosis in heart failure with ejection fraction retention (HFrEF) [27]. Other evidence suggests that CCL17 aggravates myocardial injury by suppressing recruitment of regulatory T cells [28]. All of these demonstrate immune infiltration of peripheral blood T cells and activation of inflammatory pathways in heart failure after myocardial infarction.

The proteins encoded by FOS can form a transcription factor complex AP-1 with proteins of the Jun family through a leucine zipper. In human myocardial infarction, the expression of AP‐1was significantly increased in heart tissues, which wasvparallel to the increase in matrix metalloproteinases‐9 level [29]. DUSP1 was up-regulated after acute myocardial infarction [30]. DUSP1 is cardioprotective genes that play a critical role in the heart by dampening p38 MAPK signaling that would otherwise reduce contractility and induce cardiomyopathy [31]. CXCL8, also known as IL‐8, is major mediator of inflammatory response via its potent chemotaxis for neutrophils. Previous studies have demonstrated that the serum level of IL‐8 after percutaneous intervention may serve as a predictor of the HF development in patients with myocardial infarction [32]. IKK/NF-κB activation in cardiomyocytes is sufficient to cause cardiomyopathy and heart failure by inducing an excessive inflammatory response and myocyte atrophy [33]. We proved FOS, DUSP1, CXCL8, and NFKBIA were four potential biomarkers to distinguish HF with non-HF patients.

For this exploratory study, we utilized publicly available data from different sources. Although reproducing the results in difference cohorts may also be a strength, we should remain cautious when interpreting these results. Some studies used PBMCs (which do not contain neutrophils), while other used whole blood samples, and combining data from different arrays can affect the reliability of the results. In addition, the definitions of AMI and HF differ between data sets or between studies, which may affect the reliability of the results. Finally, future studies should assess the biomarker potential (eg. sensitivity and specificity) and clinical potential of the identified transcripts in large patient populations [34].

## Supplementary Material

Supplementary Figure S1 and Tables S1-S7

## Data Availability

The datasets used and/or analysed during the current study are available from the corresponding author on reasonable request.

## Abbreviations

AMI: acute myocardial infarction
BNP: brain natriuretic peptide
CHD: coronary heart disease
CMP: Common Myeloid Progenitors
HF: heart failure
NK: natural killer
RT-qPCR: quantitative reverse transcription-PCR
TNT: troponin T
