# Supplementary material for: Identification of biomarkers and immune infiltration in acute myocardial infarction and heart failure by integrated analysis
Source: Biosci Rep. 2023 Jul 7;43(7):BSR20222552. doi: 10.1042/BSR20222552 (PMC10329185; doi:10.1042/BSR20222552)
Supplement: Supplementary Figure S1 and Tables S1-S7 [file BSR-2022-2552_supp.pdf]

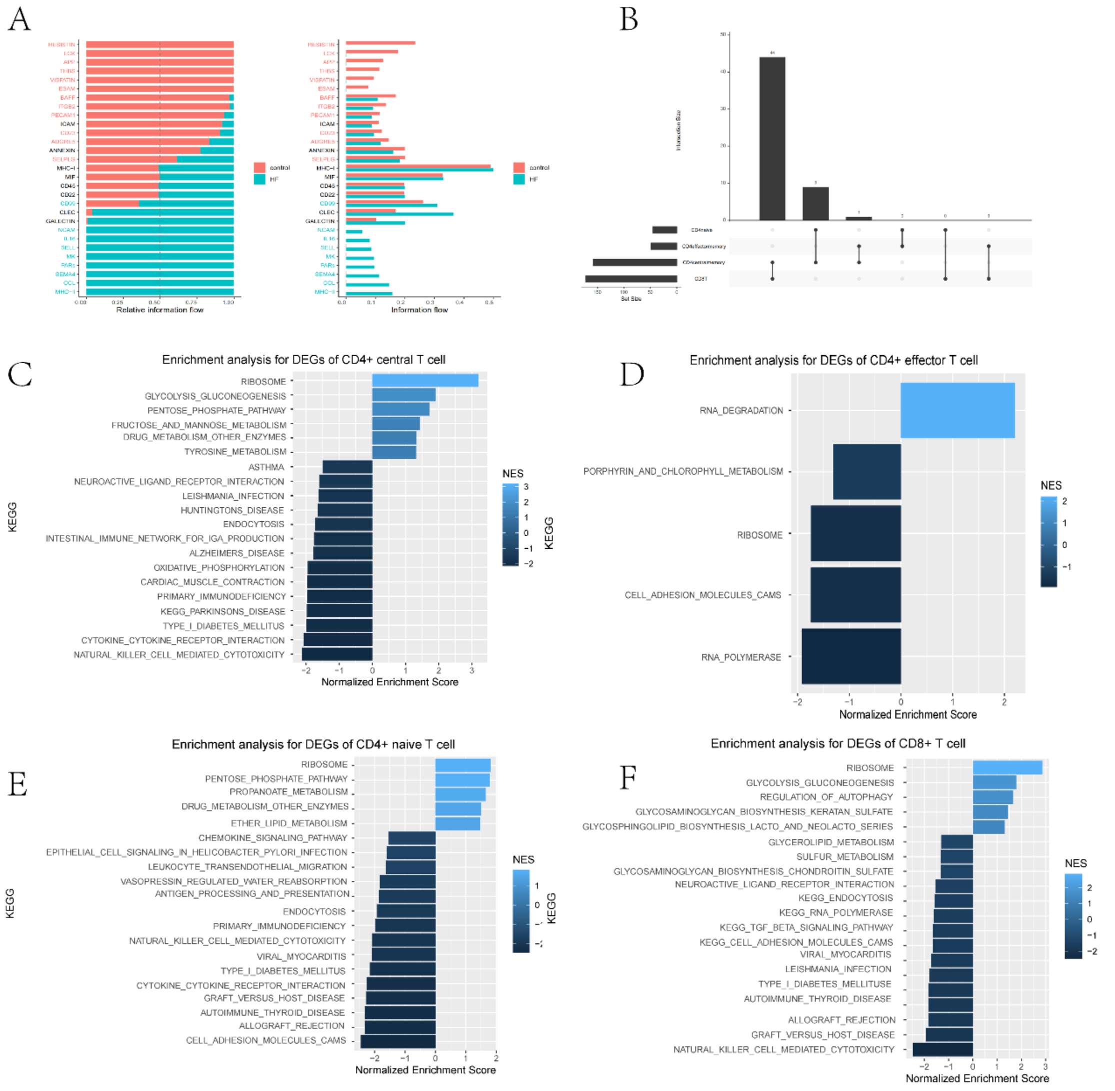

Figure Legends: (A) Enriched pathways in control and heart failure group by “Cellchat” R package. (B) Differently expressed genes in four T cell subsets between control and heart failure group was calculated by “limma” package (abs.logFC>0.5 & P.value<0.05). An intersection of four gene sets in four T cell subsets (CD4+ central memory T cells, CD4+ effector memory T cell, naïve CD4+ T cell and CD8+ T cell) was visualized by upset plot. (C-F) enrichment analysis for the DEGs of four kinds of T immune cells.

Table S1 Real time PCR primers

Primers	Sequences (5'–3')
FOS Forward	GGCTTCCCTTGATCTGACT
FOS Reverse	ATGCTCTTGACAGGTTCCA
DUSP1 Forward	CGGAATCTGGGTGCAGTT
DUSP1 Reverse	TGCGGGAAGCGTGATAC
CXCL8 Forward	GTGCTGTGTTGAATTACGGA
CXCL8 Reverse	TTGACTGTGGAGTTTTGGC
NFKBIA Forward	GCACCTCCACTCCATCC
NFKBIA Reverse	ATCAGCACCCAAGGACAC
CEBPD Forward	GAGCCTGCGCCCTTCTA
CEBPD Reverse	TCTCGTCGTCGTACATGGC
BCL2A1 Forward	CCAGGCAGAAGATGACAGA
BCL2A1 Reverse	TGGACTGAGAACGCAACA
SAMSN1 Forward	GGATGAGGAAGATGGAGAGA
SAMSN1 Reverse	GCTTGTTATGCCACTTGATG
GAPDH Forward	ACAACTTTGGTATCGTGGAAGG
GAPDH Reverse	GCCATCACGCCACAGTTTC

Table S2: The proportion results of 33 kinds of immune cells obtained by xcell analysis between CHD and AMI group.

	All<br>N=199	AMI<br>N=139	CHD<br>N=60	P Value
aDC	0.01 (0.01)	0.01 (0.01)	<0.01 (<0.01)	0.001
B-cells	0.04 (0.03)	0.03 (0.03)	0.04 (0.03)	0.186
Basophils	0.01 (0.01)	0.01 (0.02)	<0.01 (0.01)	0.006
CD4+ memory T-cells	0.02 (0.02)	0.02 (0.02)	0.02 (0.02)	0.001
CD4+ naive T-cells	0.01 (0.01)	0.01 (0.01)	0.01 (0.01)	0.761
CD4+ T-cells	0.02 (0.02)	0.02 (0.02)	0.02 (0.02)	0.207
CD4+ Tcm	0.04 (0.03)	0.04 (0.03)	0.03 (0.02)	0.109
CD4+ Tem	0.02 (0.02)	0.02 (0.02)	0.02 (0.02)	0.159
CD8+ naive T-cells	0.01 (0.01)	0.01 (0.01)	0.01 (0.01)	0.039
CD8+ T-cells	0.08 (0.04)	0.07 (0.04)	0.11 (0.03)	0.000
CD8+ Tcm	0.06 (0.04)	0.05 (0.03)	0.08 (0.03)	0.000
CD8+ Tem	<0.01 (0.01)	<0.01 (<0.01)	0.01 (0.01)	0.000
cDC	0.02 (0.02)	0.02 (0.02)	0.01 (0.01)	0.006
Class-switched memory B-cells	0.01 (0.01)	0.01 (0.01)	0.01 (0.01)	0.474
DC	<0.01 (<0.01)	<0.01 (<0.01)	<0.01 (<0.01)	0.023
Eosinophils	<0.01 (<0.01)	<0.01 (<0.01)	<0.01 (<0.01)	0.065
iDC	<0.01 (<0.01)	<0.01 (<0.01)	<0.01 (<0.01)	0.960
Macrophages	0.01 (0.01)	0.01 (0.01)	<0.01 (<0.01)	0.000
Macrophages M1	<0.01 (<0.01)	<0.01 (<0.01)	<0.01 (<0.01)	0.000
Macrophages M2	0.01 (<0.01)	0.01 (0.01)	<0.01 (<0.01)	0.000
Mast cells	<0.01 (<0.01)	<0.01 (<0.01)	<0.01 (<0.01)	0.003
Memory B-cells	0.01 (0.01)	0.01 (0.01)	0.01 (0.01)	0.655
Monocytes	0.01 (0.01)	0.01 (0.01)	<0.01 (<0.01)	0.000

	All N=199	AMI N=139	CHD N=60	P Value
naive B-cells	0.01 (0.01)	0.01 (0.01)	0.02 (0.01)	0.037
Neutrophils	<0.01 (<0.01)	<0.01 (<0.01)	<0.01 (<0.01)	0.094
NK cells	0.03 (0.03)	0.02 (0.02)	0.05 (0.03)	0.000
NKT	0.04 (0.03)	0.05 (0.03)	0.02 (0.02)	0.000
pDC	<0.01 (<0.01)	<0.01 (<0.01)	<0.01 (<0.01)	0.542
pro B-cells	<0.01 (<0.01)	<0.01 (<0.01)	<0.01 (<0.01)	0.017
Tgd cells	<0.01 (<0.01)	<0.01 (<0.01)	<0.01 (<0.01)	0.451
Th1 cells	<0.01 (<0.01)	<0.01 (<0.01)	<0.01 (<0.01)	0.773
Th2 cells	0.02 (0.02)	0.01 (0.02)	0.03 (0.02)	0.000
Tregs	0.01 (0.01)	0.01 (0.01)	0.01 (0.01)	0.022

Table S3: 150 DEGs between CHD and AMI group.

Gene name	log2FC	adj.P.Val
EDA2R	0.518848786	2.31E-27
ASGR2	0.854140664	1.86E-25
SOCS3	1.244665538	8.46E-23
STAB1	0.882702593	1.97E-22
ARAP2	-0.526479385	1.97E-22
TSPO	0.624479226	9.52E-22
TBC1D2	0.539395253	1.32E-21
AQP9	0.989527153	6.31E-21
ECRP	1.067485792	1.07E-20
KPNA5	-0.537607759	1.07E-20
ODF2L	-0.632747445	1.22E-19
SIGLEC7	0.510018474	1.63E-19
ST14	0.708030503	2.38E-19
VPS13A	-0.525412537	2.91E-19
S100A9	0.678166197	3.28E-19
CD14	0.569738805	6.30E-19
MCEMP1	0.626841851	6.74E-19
BLVRB	0.531746173	9.53E-19
LRP1	0.532735531	1.20E-18
FAM20C	0.575672691	1.53E-18

CEP78	-0.710153418	1.84E-18
MYBL1	-0.757397348	2.03E-18
NFE2	0.634846682	3.90E-18
TFDP2	-0.538521478	6.33E-18
LILRB4	0.587349217	7.22E-18
TMEM150B	0.563572062	7.42E-18
DYSF	0.82681863	1.93E-17
GPR162	0.543416044	3.01E-17
PLB1	0.503562433	3.60E-17
ADM	0.601412685	4.63E-17
CLEC2D	-0.553355767	4.63E-17
SNORD45C	-0.554097883	1.16E-16
CYP1B1	0.773116571	3.83E-16
SLC4A4	-0.580275908	5.88E-16
PPARG	0.768385762	6.80E-16
RNASE2	0.822415366	7.16E-16
FLVCR2	0.570809477	7.88E-16
SNORD59B	-0.548718382	9.17E-16
TCN2	0.793618805	1.08E-15
CSF3R	0.518986107	1.52E-15
SIGLEC9	0.563907982	1.72E-15
PRSS23	-0.589994121	2.02E-15

FAM169A	-0.53623388	2.14E-15
TBC1D19	-0.521053183	2.26E-15
RNASE1	0.808168031	2.41E-15
SASH1	0.591912652	3.50E-15
SNORD81	-0.59200875	3.56E-15
SIGLEC16	0.762246478	4.61E-15
SCARNA9	-0.694200097	4.92E-15
HRH2	0.506552005	5.05E-15
LILRA5	0.557588642	5.16E-15
C12orf75	-0.627780574	6.22E-15
WAS	0.507298084	6.49E-15
CR1	0.637276968	8.41E-15
CCR1	0.628921206	1.06E-14
THBD	0.524208029	1.06E-14
SNORD30	-0.634441114	1.10E-14
SYNE2	-0.50748927	1.68E-14
KLRD1	-0.696506941	2.26E-14
CYP27A1	0.648748064	2.27E-14
SNORD54	-0.693628638	3.05E-14
SLC11A1	0.500541137	3.40E-14
RASGRP4	0.510760065	5.72E-14
SNORD28	-0.626810965	1.25E-13

PDGFD	-0.641957445	1.60E-13
SNORD58A	-0.597397294	1.62E-13
MAFB	0.50144835	3.90E-13
DTHD1	-0.739923167	4.27E-13
RORA	-0.506179768	5.79E-13
C1orf21	-0.556302663	6.00E-13
SH2D1A	-0.596933582	6.04E-13
SNORD61	-0.696086652	6.36E-13
MERTK	0.715316407	1.05E-12
SYTL2	-0.565150334	1.25E-12
SAMD3	-0.539529152	1.30E-12
FAM20A	0.975637792	1.48E-12
CD226	-0.531116374	1.49E-12
JAKMIP2	-0.568038584	1.79E-12
CCR2	0.524042894	1.81E-12
SNORD50B	-0.523342212	1.81E-12
KLRA1P	-0.715378983	2.14E-12
KCNJ15	0.57478067	2.20E-12
TGFBR3	-0.574007343	3.06E-12
KLRG1	-0.67424992	3.22E-12
RNU5A.1	-0.562806507	3.85E-12
GBP5	-0.506373677	4.01E-12

BIRC3	-0.518290118	4.32E-12
KLRC1	-0.711877171	6.11E-12
HP	1.117553682	6.12E-12
GBP4	-0.503322257	7.31E-12
MYO6	-0.568917493	7.55E-12
SNORD50A	-0.654016583	1.06E-11
SLCO4C1	-0.560747316	1.57E-11
MIR21	0.683029153	1.78E-11
ACSL1	0.545405825	1.79E-11
S100A12	0.520325164	1.80E-11
SNORD82	-0.67540826	2.21E-11
VSIG4	0.730317193	2.34E-11
MIR223	0.54465773	3.10E-11
HIST1H4C	-0.536719605	4.25E-11
1-Mar	0.532323368	4.36E-11
CPA3	-0.531660966	5.00E-11
CCL4	-0.528869629	5.91E-11
SCARNA6	-0.590965498	6.66E-11
ZNF737	-0.519223329	7.72E-11
PADI2	0.64521517	8.48E-11
TARP	-0.544410356	8.62E-11
C1QC	0.507427717	1.39E-10

KLRC4.KLRK1	-0.867661332	1.40E-10
SNORA20	-0.805198981	1.53E-10
IL18RAP	-0.600044883	1.57E-10
STEAP4	0.582451097	4.10E-10
KLRF1	-0.713289846	4.65E-10
SERPINB2	0.634714474	4.76E-10
SLED1	0.7345719	1.11E-09
SNORD105	-0.654190575	1.13E-09
CES1P1	0.696684489	1.14E-09
TRIB1	0.597946527	1.17E-09
SH3PXD2B	0.508917067	1.20E-09
SNORA75	-0.528370004	1.24E-09
MS4A3	-0.73659473	1.35E-09
CD163	0.620461013	1.60E-09
SNORD15B	-0.520587609	1.60E-09
KLRC3	-0.763869257	1.88E-09
EPB41L4A	-0.53358453	2.03E-09
FPR2	0.555217711	2.42E-09
NRG1	0.696282495	2.85E-09
FMN1	0.71791379	3.04E-09
DSC2	0.58263112	3.52E-09
HRH4	-0.606171735	3.67E-09

CD160	-0.570966097	3.68E-09
CES1	0.757620039	5.27E-09
LILRA3	0.577844509	6.34E-09
MS4A4A	0.59921267	1.45E-08
SNORD20	-0.69617947	7.08E-08
FGFBP2	-0.544684734	1.57E-07
CLC	-0.923517185	3.19E-07
TNFAIP6	0.66631651	6.04E-07
IFITM3	0.523880752	7.32E-07
SERPINB10	0.576035162	1.19E-06
SNORA24	-0.563849116	5.30E-06
HBEGF	0.548408108	5.81E-06
SNORD60	-0.501440589	2.10E-05
IL1R2	0.573856664	3.49E-05
FOLR3	0.623946833	6.17E-05
TMEM176A	0.822925668	0.000163652
EGR1	0.747064812	0.000386595
EGR2	0.63984473	0.001176634
TMEM176B	0.577327177	0.002588821
ADGRG7	-0.62826879	0.008867036

Table S4 1: The proportion results of 33 kinds of immune cells obtained by xcell analysis between non-HF and HF group.

	All<br>N=97	non-HF<br>N=73	HF<br>N=24	p.overall
aDC	<0.01 (0.02)	<0.01 (0.02)	<0.01 (<0.01)	0.936
B-cells	0.02 (0.01)	0.02 (0.01)	0.01 (0.02)	0.096
Basophils	0.03 (0.05)	0.03 (0.04)	0.03 (0.05)	0.760
CD4+ memory T-cells	0.03 (0.02)	0.03 (0.02)	0.02 (0.01)	0.016
CD4+ naive T-cells	<0.01 (<0.01)	<0.01 (<0.01)	<0.01 (<0.01)	0.316
CD4+ T-cells	0.02 (0.02)	0.03 (0.02)	0.01 (0.01)	0.004
CD4+ Tcm	<0.01 (<0.01)	<0.01 (<0.01)	<0.01 (<0.01)	0.702
CD4+ Tem	0.11 (0.04)	0.11 (0.03)	0.09 (0.03)	0.004
CD8+ naive T-cells	<0.01 (<0.01)	<0.01 (0.01)	<0.01 (<0.01)	0.000
CD8+ T-cells	<0.01 (<0.01)	<0.01 (0.01)	<0.01 (<0.01)	0.018
CD8+ Tcm	<0.01 (<0.01)	<0.01 (<0.01)	<0.01 (<0.01)	0.134
CD8+ Tem	<0.01 (<0.01)	<0.01 (<0.01)	<0.01 (<0.01)	0.120
cDC	0.01 (0.02)	0.01 (0.02)	0.01 (0.01)	0.694
Class-switched memory B-cells	<0.01 (<0.01)	<0.01 (<0.01)	<0.01 (<0.01)	0.105
DC	<0.01 (<0.01)	<0.01 (<0.01)	<0.01 (<0.01)	0.130
Eosinophils	<0.01 (<0.01)	<0.01 (<0.01)	<0.01 (<0.01)	0.703
iDC	0.01 (0.06)	0.01 (0.06)	0.02 (0.05)	0.321
Macrophages	0.02 (0.01)	0.01 (0.01)	0.02 (0.01)	0.257
Macrophages M1	<0.01 (<0.01)	<0.01 (<0.01)	<0.01 (<0.01)	0.563
Macrophages M2	0.01 (<0.01)	0.01 (<0.01)	0.01 (<0.01)	0.517
Mast cells	<0.01 (<0.01)	<0.01 (<0.01)	<0.01 (<0.01)	0.422
Memory B-cells	0.01 (0.01)	0.01 (0.01)	0.01 (0.01)	0.485
Monocytes	<0.01 (<0.01)	<0.01 (<0.01)	<0.01 (<0.01)	0.533

	All	non-HF	HF	p.overall
	N=97	N=73	N=24	
naive B-cells	<0.01 (<0.01)	<0.01 (<0.01)	<0.01 (<0.01)	0.249
Neutrophils	<0.01 (<0.01)	<0.01 (<0.01)	<0.01 (<0.01)	0.242
NK cells	<0.01 (<0.01)	<0.01 (<0.01)	<0.01 (<0.01)	0.646
NKT	0.02 (0.02)	0.01 (0.02)	0.02 (0.03)	0.293
pDC	<0.01 (<0.01)	<0.01 (<0.01)	<0.01 (<0.01)	0.129
pro B-cells	<0.01 (0.02)	<0.01 (0.02)	<0.01 (<0.01)	0.856
Tgd cells	<0.01 (<0.01)	<0.01 (<0.01)	<0.01 (<0.01)	0.143
Th1 cells	0.01 (0.01)	0.01 (0.01)	0.01 (0.01)	0.257
Th2 cells	0.02 (0.05)	0.01 (0.04)	0.04 (0.07)	0.856
Tregs	<0.01 (<0.01)	<0.01 (<0.01)	<0.01 (<0.01)	0.803

Data are presented as mean (SD). And all results are calculated by wilcoxon rank-sum test.

Table S5: The proportion of multiple immune cell subsets between control and post-MI HF group.

immune cell	Control (n=1974)	post-MI HF (n=18355)
Memory B cell	0.105369807	0.026259875
Naive B cell	0.125126646	0.029746663
CMP	0.014184397	0.001253065
Monocyte:CD16-	0.168186424	0.02195587
NK cell	0.042046606	0.026096431
T_cell:CD4+_central_memory	0.333839919	0.244565513
T_cell:CD4+_effector_memory	0.006079027	0.118060474
T_cell:CD4+_Naive	0.022796353	0.203650232
T_cells:CD8+	0.182370821	0.328411877

Table S6: 59 DEGs between non-HF and HF group.

Gene name	log2FC	adj.P.Val
ARG1	1.112852962	0.029943354
VNN1	1.108081203	0.02406228
FOLR3	1.017308043	0.03712918
SAMSN1	0.994807635	0.018500276
BMX	0.959422602	0.007822286
QPCT	0.93133939	0.00322543
TPST1	0.748775997	0.019640107
BCL2A1	0.732644043	0.032507257
FOS	0.729216856	0.005411351
CSTA	0.713754823	0.029554873
DYSF	0.701005825	0.008734577
CYP1B1	0.695758844	0.033736619
IGF2R	0.695549685	0.008351239
PYGL	0.690627917	0.00847515
SLC37A3	0.68726698	0.005411351
ECHDC3	0.669144806	0.006984884
SDCBP	0.652687046	0.006652084
SAP30	0.645643169	0.012136919
VNN3	0.643403596	0.036747285
NQO2	0.642883316	0.006871594

PLAUR	0.636856609	0.009826509
HMGB2	0.636433388	0.011949994
PGD	0.635225235	0.00653293
F5	0.633339725	0.034267886
IFRD1	0.629479203	0.01329506
DUSP1	0.627483889	0.043093059
MEGF9	0.626150967	0.016988138
GPR160	0.616150938	0.004525612
AQP9	0.613838529	0.030273336
ZNF281	0.603739945	0.004274962
LBR	0.599455438	0.005463037
ANKRD22	0.59794682	0.017309577
RTN3	0.59201302	0.005411351
IL8	0.584760829	0.012634343
C20orf24	0.577551186	0.002608692
H3F3B	0.569284102	0.004274962
HK2	0.564510662	0.001794128
PRKAR1A	0.560396614	0.001794128
LRG1	0.555301316	0.036747285
SRPK1	0.554129478	0.023660742
ABHD5	0.553761388	0.034267886
CEBPD	0.550622158	0.001704084

RALB	0.550010008	0.008031104
NCF4	0.5481649	0.037429146
S100P	0.545558689	0.019544825
FRAT2	0.539268669	0.008559834
MAN2A2	0.537720662	0.012041428
IFNGR2	0.535822355	0.001794128
NFIL3	0.531564275	0.047864825
BPI	0.53066909	0.030480512
NFKBIA	0.530282049	0.031125326
ABTB1	0.523604573	0.012883438
MAP2K6	0.518514738	0.012695838
PXK	0.513290768	0.034184306
GCA	0.508606172	0.033050454
GABARAPL1	0.500172763	0.011512179
LGALS2	-0.513250839	0.004274962
CDC25B	-0.596178496	0.006265975
CD3D	-0.618635719	0.028816748

Table S7: Baseline characteristics for HF and non-HF group

	All<br>N=60	Non-HF<br>N=40	HF<br>N=20	P Value
STEMI (%)	33 (55.00%)	16 (40.00%)	17 (85.00%)	0.002
Male (%)	49 (81.67%)	32 (80.00%)	17 (85.00%)	0.736
Age (Years)	60.35 (10.51)	58.55 (10.66)	63.95 (9.46)	0.052
Hypertension (%)	22 (36.67%)	10 (25.00%)	12 (60.00%)	0.018
Hypercholesterolemia (%)	29 (48.33%)	13 (32.50%)	16 (80.00%)	0.001
Diabetes (%)	9 (15.00%)	2 (5.00%)	7 (35.00%)	0.004
Current smoker (%)	28 (46.67%)	16 (40.00%)	12 (60.00%)	0.234
Previous smoker (%)	14 (23.33%)	11 (27.50%)	3 (15.00%)	0.347
Previous MI (%)	5 (8.33%)	2 (5.00%)	3 (15.00%)	0.322
EF (%)	52.07 (10.38)	58.60 (3.26)	39.00 (6.59)	<0.001
BMI (kg/m^2)	26.97 (4.32)	26.68 (4.84)	27.54 (3.02)	0.401
Peak TnT (ug/L)	2.82 (2.52)	1.37 (1.39)	5.73 (1.55)	<0.001
Peak CRP (mg/L)	38.52 (51.70)	15.22 (17.97)	85.11 (64.89)	<0.001
WBC (10^9/L)	10.13 (3.42)	8.95 (2.68)	12.48 (3.56)	<0.001
Granulocyte Abs (10^9/L)	6.70 (2.96)	5.82 (2.51)	8.47 (3.07)	0.002
Lymphocyte Abs (10^9/L)	2.41 (0.92)	2.26 (0.96)	2.70 (0.76)	0.060
Monocyte Abs (10^9/L)	0.78 (0.39)	0.63 (0.20)	1.07 (0.50)	0.001

Data are presented as mean (SD), or median (percentile). Abbreviations: HF: heart failure; STEMI: ST segment Elevation Myocardial Infarction; EF: Ejection Fraction; BMI: body mass index; TnT: troponin T; CRP: C-reactive protein; Abs: absolute counting.
